# Probe-Based Fluorescence Spectroscopy for In Situ Brain Tumor Measurements During Resection and Needle Biopsies

**DOI:** 10.3390/biomedicines13030537

**Published:** 2025-02-20

**Authors:** Karin Wårdell, Elisabeth Klint, Johan Richter

**Affiliations:** 1 Department of Biomedical Engineering, Linköping University, 581 85 Linköping, Sweden; elisabeth.klint@liu.se (E.K.); johan.richter@regionostergotland.se (J.R.); 2 Department of Neurosurgery, Linköping University Hospital, 581 85 Linköping, Sweden

**Keywords:** five aminolaevulinic acid (5-ALA), brain tumor, fluorescence, laser Doppler flowmetry (LDF), neuronavigation, protoporphyrin IX (PpIX), resection, stereotactic

## Abstract

**Background/Objectives**: Primary brain tumors are difficult to identify intraoperatively due to their infiltrative character in the marginal zone. Several optical methods have been suggested. Of these, 5-ALA-induced fluorescence visualized through a microscope is the most common. The aim is to present an investigational probe-based optical system and its translation for clinical use, summarize previous studies, and give examples of clinical implementations during resection and burr hole biopsies. **Methods**: The FluoRa system combines 5-ALA fluorescence spectroscopy with laser Doppler flowmetry (LDF). Probe designs are available for brain tumor resection (hand-held probe) or burr hole needle biopsies (frame-based or navigated). The outer cannulas of biopsy needles are modified with an opening at the tip for simultaneous use with optical probes during insertion along the trajectory. An updated version of FluoRa is introduced and experimentally investigated. **Results**: Probe-based fluorescence spectroscopy has been successfully translated for clinical use and applied during brain tumor resection (n = 75) and burr hole needle biopsies (n = 47). Forward-looking optical measurements through the biopsy needle reduce the number of trajectories (28/27) compared to prior to insertion (28/20), at the same time that the target for tissue sampling can be identified in situ. Additionally, increased microcirculation is identified along the trajectory with LDF. This is accomplished with FluoRa. **Conclusions**: Intraoperative probe-based spectroscopic measurements quantify 5-ALA fluorescence and thus identify glioblastoma and lymphoma tissue in situ during resection and burr hole needle biopsies.

## 1. Introduction

Identifying primary brain tumors during resection is important due to their infiltrative character in the marginal zone, which is a difficult task, even for an experienced neurosurgeon. The first technical principle for the protoporphyrin IX (PpIX) microscope visualization of tumor tissue was presented by Stummer et al., in 1998 [1]. In the last decade, blue-light microscopes visualizing five aminolaevulinic acid (5-ALA)-induced PpIX fluorescence have become standard equipment in many neurosurgical clinics in Europe. In 2017, the fluorescence microscopes were FDA approved and introduced in the USA for fluorescence-guided resection (FGR) [2].

Additionally, other optical techniques for brain tumor identification have been suggested and are under development [3]. Examples are fluorescein sodium fluorescence [4], indocyanine-green fluorescence [5], flow cytometry [6], hyperspectral imaging [7], and Raman spectroscopy and imaging [8,9]. A major advantage of PpIX fluorescence as an indicator for glioblastoma is that it is well-documented [10,11] and also clinically recognized [2]. In addition to glioblastoma, PpIX fluorescence has been utilized for lymphoma [12,13], intracranial meningioma [14,15], and occasionally also for metastases [16,17]. The utility of the blue-light microscope has also been demonstrated in relation to needle biopsies. By placing the tissue sample under the microscope, the presence of PpIX fluorescence is visually inspected [18,19]. This method, however, does not identify malignant tissue in situ. Another drawback is subjective reading, which is based on the neurosurgeon’s naked eye assessment of the microscope view at the same time that the fluorescence is bleached.

The question of applying PpIX fluorescence during needle biopsies was previously addressed in 2002 by Backlund and colleagues. Using a stereotactic technique, glioblastoma was identified intraoperatively through an optical fiber connected to a spectrometer system [20]. Due to this being a very bulky device with two laser sources, it was only used in a limited number of patients. It was first replaced by a miniature system, easy to handle in the operating room (OR) but with a fluorescent signal that was very weak to noise due to the use of a diode as light source [21]. Based on the knowledge from these initial studies, our research group developed a portable fluorescence device. Photobleaching was investigated and system parameter settings optimized. Further technical problems like interference from surrounding operating lamps were overcome before the Linköping system was introduced for measurements during brain tumor resection [22].

Our group has, in parallel to the development of the fluorescence technique for brain tumor resection, invented a method for intraoperative guidance during deep brain stimulation (DBS) implantation. It is based on laser Doppler flowmetry (LDF) and allows for in situ forward-looking measurements of microcirculation and gray-whiteness [23]. For this reason, it has been used in more than 130 DBS implantations [24]. It became natural to combine fluorescence spectroscopy with LDF. First, two separate systems were joined via the same fiber optical probe [25]. As a second step, the functions were built into one device, FluoRa [26], that simultaneously can identify malignant tissue objectively, act as a “vessel alarm”, and in high resolutions, distinguish between shades of gray and white matter in situ.

The overall aim of this paper is to present our optical systems and fiber optical probes for tumor surgery and to give examples of applications in open brain tumor resection and burr hole needle biopsies. The latest version of our system is introduced. A specific aim was to investigate the measurement time, intraoperative response time, and number of used trajectories during needle biopsies performed with optical guidance.

## 2. Materials and Methods

In this section, the measurement systems are described, and examples of applications are given for the identification of malignant brain tumor tissue intraoperatively. All studies presented in this paper were approved by the Ethics Committee (EPN-2015-138-32; EPM-2020-01404) and the patients gave their informed written consent prior to inclusion.

### 2.1. FluoRa: A System for Fluorescence, Microcirculation, and Grey-White Matter Measurements

**Figure 1:**
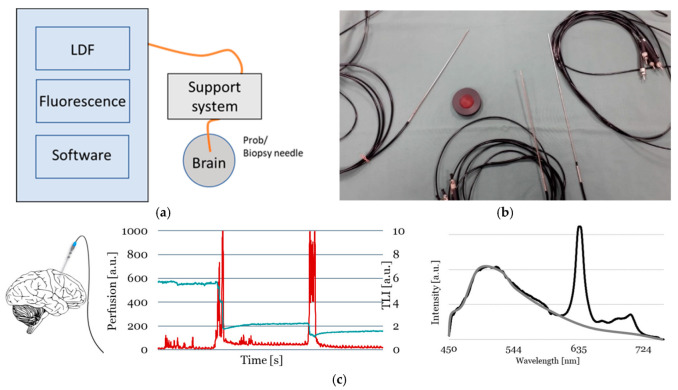
(**a**) A schematic overview of the FluoRa systems. (**b**) A fluorescence reference plate surrounded by typical optical probes. (**c**) Measurement examples of Perfusion (red) and total light intensity (TLI, dark blue) in the middle panel. The large red spikes are caused by the movement of the probe to a new measurement site. The Perfusion signal is immediately stabilized after the movement and the heartbeats visible in the microcirculation. Fluorescent spectrum with PpIX peak (black) and without (gray) are presented to the right. a.u. = arbitrary units.

FluoRa is an in-house developed investigational system designed for use during neurosurgery. The system comprises a measurement box, a laptop, fiber optical probes, and modified biopsy needles. The measurement box has a fluorescence module which is used for the intraoperative identification of glioblastoma after the administration of 5-ALA. The second feature of FluoRa is the incorporation of LDF for the simultaneous measurement of the cerebral microcirculation (denoted Perfusion) and the gray-whiteness of the cerebral tissue (denoted total light intensity, TLI). In its original version, FluoRa 1.0, the system comes with a separate, analog LDF unit [26,27]. In the updated version, FluoRa 2.0, presented in this paper (see Section 2.2), a digital LDF unit is built into the measurement box. Both systems are controlled by software using a similar user interface and are also compatible with the same set of optical probes. Figure 1 shows a schematic overview of the FluoRa systems.

#### 2.1.1. Optical Probes

The probes to the FluoRa systems are in-house designed and manufactured. They are built with biocompatible materials and withstand repetitive sterilizations using the STERRAD^®^ procedure [28]. The probes consist of an end part made of stainless steel, a 5 m long cable with optical fibers inside, and SMA and ST fiber optic connectors for coupling to the fluorescence and LDF parts of the measurement box, respectively (Figure 1b). The end part is in contact with the tissue and can have the shape of a handle or matched dimensions for supportive navigation systems and biopsy needles. A transmitting and receiving fiber pair for the fluorescence and LDF parts are placed next to each other in the stainless-steel tube where they are fixed by biocompatible glue. The optical fibers for the fluorescence part have a diameter of 200 μm and the numerical aperture (NA) is 0.22. The LDF fibers are thinner with a diameter of 125 μm and a broader numerical aperture (NA = 0.37). The fiber optical tips are smoothly polished for optimal light interaction with the tissue. The following probe designs are presently available:

- Hand-held probe for open surgery;
- Probes for modified biopsy needles;
- Probes for guidance and the creation of a trajectory.

Depending on the outer dimension and optical fiber configuration of the different probes, the system can be used during resection for the investigation of a tumor’s marginal zone in open surgery [29] or for burr hole needle biopsies with a real-time in situ identification of malignant tissue suitable for sampling [25]. Probes are also available to create a trajectory prior to insertion of a DBS electrode, i.e., as a guide for the actual DBS-lead implant [24].

The long fiber optical cable together with an isolation unit in the measurement box ascertains that the system fulfills the electric safety requirements. Laser safety is accomplished by widening the laser beam and using a shutter, which is closed except for the seconds the fluorescence spectrum is sampled. For a detailed description of FluoRa 1.0, see [26].

#### 2.1.2. Modified Biopsy Needles

To be able to perform optical measurements during the insertion of a biopsy needle towards a brain tumor, the outer cannula of the biopsy needle must be able to transmit the light to the tissue through an opening at the tip. Two sets of biopsy needle kits have been modified. The Sedan Side-Cutting Biopsy Kit (Elekta Instrument AB, Stockholm, Sweden) [30] to be used together with the Leksell Stereotactic System (LSS, Elekta AB, Stockholm, Sweden) and the Passive Biopsy Needle which is designed to work with a frameless neuronavigation system (StealthStation S8, Medtronic Inc., Minneapolis, MN, USA) [27].

**Figure 2:**
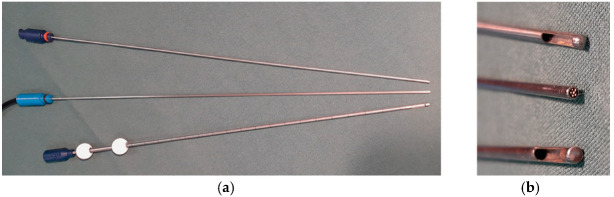
(**a**) The Passive Biopsy Needle modified with an opening at the tip of the outer cannula. The related optical probe is placed between the outer and inner cannulas. (**b**) From top to bottom: inner cannula, optical probe with enlighten fibers at the tip, cannula modified with an opening.

The optical probes’ lengths and diameters were designed to a perfect fit inside the outer cannulas of the respective biopsy needles, i.e., the probes have the same dimensions as the biopsy needles’ inner cannulas. The probe tips were smoothly rounded and matched to the respective cannulas’ openings. A new biopsy kit of Passive Biopsy Needle was prepared for each surgery, whereas the Sedan Side-Cutting Biopsy Kit was re-sterilized according to the manufacturer’s instructions. Figure 2 shows a modified Passive Biopsy Needle with the related optical probe, with a close-up of the tips in Figure 2b.

#### 2.1.3. Software

The software is used for the data collection, presentation, and storage of fluorescence spectra, Perfusion, and TLI signals, and is programmed in Labview 2019 (National Instruments, Austin, TX, USA) for FluoRa 1.0. The software has three measurement modes:

- Fluorescence (FL);
- Fluorescence and laser Doppler flowmetry (FL-LDF);
- Laser Doppler flowmetry (LDF).

The FL-mode is activated by the user separately and initiates three short consecutive laser pulses of which each result in a fluorescence spectrum. In the presence of malignant tissue, the resulting spectrum from the exposed tissue will contain a PpIX peak at the red, 635 nm-wavelength (λ) region. The LDF-mode is runs continuously and measures the microcirculation (Perfusion) and gray-whiteness (TLI) of the tissue as relative variations (arbitrary units, a.u.) [23]. The FL-LDF-mode combines the above features. Examples of typical Perfusion, TLI, and PpIX fluorescence spectrum are shown in Figure 1c.

### 2.2. From FluoRa 1.0 to FluoRa 2.0

As FluoRa 2.0 has not yet been approved for clinical use, examples of experimental evaluations of the device and its comparison with FluoRa 1.0 are given. Both systems have the same ultraviolet lasers (40M18H3-F-405-PE, Z-LASER GmbH, Freiburg im Breisgau, Germany) with an output wavelength of 405 nm. The spectrometer in FluoRa 2.0 is an upgraded version (AvaSpec-Mini2048CL, Avantes B.V., Lafayette, CO, USA). During a measurement, the laser sends out three consecutive laser pulses lasting for 400 ms. Each pulse is followed by the collection of a dark spectrum which is used for the subtraction of surrounding light. Following each pulse, a spectrum is presented on the screen in real-time. The output laser power can be varied in the software between 5, 10, 15, and 20 mW. A major difference between the two systems is that the LDF unit (Periflux PF 6010, Perimed AB, Järfälla, Sweden), which is digital, is built into the measurement box of FluoRa 2.0. This makes the system smaller and easier to handle. Another improvement is that data communication goes through a USB hub (FIRENEX-UHUB, Newnex, Santa Clara, CA, USA). The software for FluoRa 2.0 has the same functions as FluoRa 1.0, but is programmed in MatLab (R2022b/R2023a, MathWorks Inc., Natick, MA, USA) By compiling the software, it becomes available without the installation of MatLab. The system conformity was assessed with the same probe connected to FluoRa 1.0 and FluoRa 2.0 using the following:

- A reference plate with fluorescent material (Figure 1b);
- Microsphere solution;
- Fingertip;
- Forearm skin after the application of ALA cream.

All four laser settings were tested with both systems on the reference plate and the shape and maximum intensity of respective fluorescence spectrum noted. To test the LDF features, the probe was positioned in a standard microsphere solution (PF1001 Motility, Perimed AB, Järfälla, Sweden). Thereafter, a measurement was carried out with the probe placed on the tip of middle finger of a healthy volunteer (Male, 59 years).

**Figure 3:**
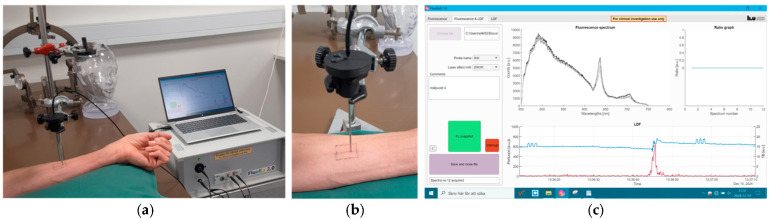
(**a**) The FluoRa 2.0 system during measurement on the forearm skin with ALA cream. (**b**) The close-up shows the probe placed in the holder and positioned lightly on the skin. (**c**) The FluoRa 2.0 user interface with data from the skin measurement. Three PpXI peaks are shown on the upper part of the panel. The Perfusion (red curve) and TLI (blue curve) signals are presented in the lower panel. The peaks in the red curve are due to the movement of the probe to a new position on the skin. The three small, squared peaks in the TLI signal appear during the three consecutive fluorescence measurements.

A final investigation was performed on the forearm of the volunteer after the topical application of Metylaminolevulinat cream (METVIX^®^ 160 mg/g, Photocure ASA, Oslo, Norway) (Ethics approval: M139-07/T83-09). Using the ALA cream mimics a neurosurgical situation as the same type of PpIX peak is expected. The cream was applied to a skin area covering approximately 2.5 cm × 2.5 cm. To avoid light exposure, the skin was covered with a dressing (Tegaderm™, 3M Health Care, Kamen, Germany) and a nontransparent cloth before the experiment was initiated. Four hours after the application, fluorescence measurements were performed at 5, 10, 15, and 20 mW at the same time as the Perfusion and TLI were recorded. Different skin spots were used for each measurement to avoid the photobleaching effect at the measurement site. Figure 3 shows the experimental setup with FluoRa 2.0 during the skin measurements with ALA cream. The probe is placed in a holder and the tip of the probe touches the skin during the recording.

### 2.3. Fluorescence Identification of Malignant Brain Tumor Tissue During Resection

Fluorescence spectroscopy can be used for the intraoperative identification of glioblastomas when 5-ALA is used as exogenous fluorophore. Initially, we used a dose of 5 mg/kg body weight, but when 5-ALA became certified for FGR, the recommended dose was increased to 20 mg/kg. Before surgery, the patient is given an oral dose of 5-ALA (Gliolan, Medac, GmbH, Wedel, Germany), which after a few hours is synthesized through the heme cycle into PpIX and accumulates in the malignant brain tissue, i.e., glioblastoma or lymphoma.

When the tissue with PpIX is exposed to blue light (λ = 405 nm), fluorescence appears in the red-pink region, corresponding to a peak with a maximum size around λ = 635 nm in the fluorescence spectrum (Figure 1c). A small peak is sometimes also seen at the λ = 704 nm. The color change from blue to red is detected through the microscope during surgery, or with the spectrometer which is used in FluoRa. Using FGR, the fluorescence is graded through the microscope by the surgeon’s naked eye as “none”, “weak” or “strong”, whereas the spectroscopy method quantifies the PpIX strength and thus objectifies the collected information. The probe-based spectroscopy method can be used together with the microscope or as stand alone on the tissue surface or inside the tumor. The hand-held probe can also be connected and calibrated to the StealthStation navigation platform and used in optical or electromagnetic mode. To locate the anatomical position of the probe in relation to the tissue as visualized in the MRI, it must be registered to the neuronavigation system. The extension with four reference points is mechanically attached to the probe handle. Then, the instrument is added to the available instruments through the stipulated calibration method in the StealthStation [27].

In this paper, the studies are summarized where our fluorescence spectroscopy systems have been applied in relation to resection with and without FGR. An example using FluoRa 1.0 during tumor resection combined with the StealthStation navigation is presented.

### 2.4. In Situ Optically Guided Brain Tumor Needle Biopsy

In the same manner as during resection, 5-ALA is administered prior to a brain tumor needle biopsy. One or more trajectories toward the tumor are planned in the preoperative anatomical MRI. During a measurement, the optical probe is placed inside the outer cannula of the biopsy needle and thereafter inserted along the precalculated trajectory towards the target, i.e., the tumorous tissue. By moving the needle kit forward and stopping for about 10 s at each site (the time can be extended), the fluorescence, Perfusion and TLI are measured simultaneously. Three fluorescence spectra are captured at each site, which takes about 1.5 s. In parallel, the LDF data are streamed and displayed on the monitor. As the probe is forward-looking, increased microcirculation can warn about possible vessel structures that have not yet been touched before moving the probe to the next site along the trajectory. In case of high Perfusion, the surgeon can extend the measurement time before going to the next site or withdraw the biopsy kit. When PpIX peaks are visible, it indicates where to take the biopsy. As the side-cutting opening of the needle is a few millimeters above the tip, the biopsy kit must be adjusted to the actual region with the highest PpIX peaks along the trajectory, and a cutting spot that minimizes bleeding can be selected with the support of the Perfusion signal. Once in place and fixated, the probe is replaced by the inner cannula of the needle and the sample is taken and subsequently sent for pathological investigation.

In total, 47 patients (age 18–80 years; 15 women) have been included for in situ fluorescence-guided burr hole tumor needle biopsies at the Department of Neurosurgery, Linköping University Hospital. Preoperative MRI was used for trajectory and target planning with LSS or StealthStation. In 20 patients where the LSS was used, the optical probe was used for measurements prior to insertion of the biopsy kit [25], and in 27 patients, the insertion along the trajectory was carried out with the probe already in the outer cannula of the biopsy needle, 3 with LSS [30] and 20 with the StealthStation [31] as the navigation feature, respectively. Four additional cases performed with the StealthStation are added in this paper for the assessment of measurement time, intraoperative response time for the pathology analysis, and the number of used trajectories. All biopsy samples were intraoperatively analyzed according to the routine at the clinic. The final neuropathological diagnosis was set postoperatively according to CNS WHO 2021 [32].

## 3. Results

### 3.1. Conformity Between FluoRa Systems

**Figure 4:**
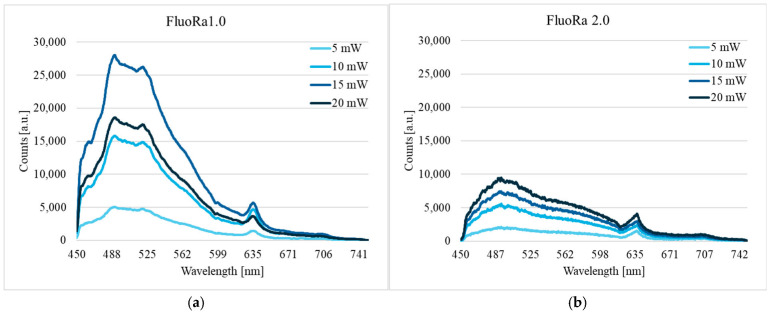
Fluorescence spectra after the application of ALA on the skin. PpIX peaks are seen at a wavelength of 635 nm. (**a**) FluoRa 1.0 and (**b**) FluoRa 2.0.

Both systems showed good agreement when measuring on the test plate (fluorescence) in Motility signals and on skin (LDF). As both systems measure relative changes, there was a small systematic difference, which was expected. There is a natural spatial variability in skin microcirculation [33], so the same values are not possible to capture. These initial measurements showed that it was feasible to record the PpIX fluorescence, Perfusion, and TLI simultaneously with FluoRa 2.0, similarly to FluoRa 1.0. The analysis of the skin recordings with ALA cream showed that the maximal intensity was about twice as high for FluoRa 1.0 compared to FluoRa 2.0, but the shape of the spectrum and PpIX appearance were similar. The spectra representing 5, 10, 15, and 20 mW from the two devices are shown in Figure 4.

### 3.2. Fluorescence-Guided Resection Using Probe-Based Spectroscopy

Table 1 summarizes the studies at Linköping University Hospital performed with our resection probe technique, with and without FGR, and with and without neuronavigation by ultrasound, LSS, and StealthStation. The probe method has been used during 75 tumor resections, of which 14 were in children [34]. The miniature system was only used during one surgical resection. Thereafter, the portable fluorescence device [22] was introduced for studies over a time period of about 10 years, before it was updated and the LDF module added, i.e., FluoRa [26].

As 5-ALA is not approved for patients under the age of 18 years, our study was performed under The Swedish Medical Products Agency (MPA) approval as a Phase II trial (Eudrat 2013-005565-40). Combined probe and FGR showed that the fluorescence methods using 20 mg/kg of 5-ALA are less suitable in children compared to adults, especially in small children, due to a weak or lack of fluorescence response. In adults, however, it has been shown that the administration of a lower 5-ALA dose (5 mg/kg) has equally reliable diagnostic performance when using the probe [36]. Moreover, this study, which included two groups of 15 patients each, indicated that the patient does not become light sensitive with the lower dose, as no PpIX peaks were detected when measuring on the patients’ forearm skin postoperatively. It must be pointed out that the investigation used the older version of the probe-based spectroscopic system [22].

**Figure 5:**
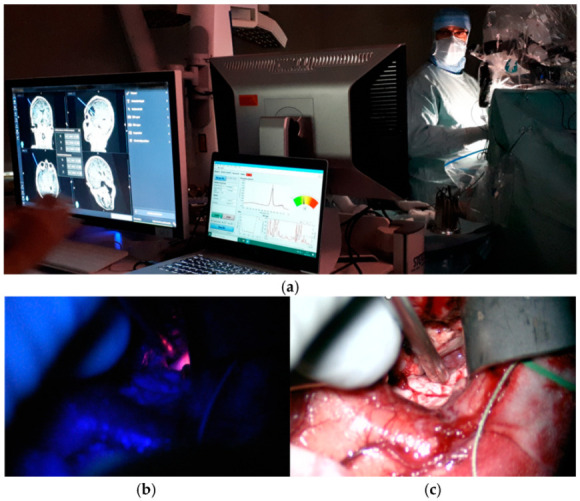
(**a**) A typical measurement situation during tumor resection where FluoRa 1.0 and the hand-held probe is used together with the blue-light microscope. The probe position is updated on the StealthStation to the left. A clear fluorescence peak is presented on the monitor in the middle. (**b**) The probe is used under the blue-light microscopy mode and PpIX fluorescence is seen as pink. In (**c**), the probe and tissue are visible in light microscopy mode. Microscopy images are snapshots from the video in ref. [29].

Our group has also shown that due to the higher sensitivity of the probe-based method, the combination of FGR and probe measurements increases the identification of malignant brain tumor tissue intraoperatively, especially along the tumor marginal zone [29]. In Figure 5, an example of a combined measurement with the blue-light microscope and the resection probe is presented together with a white light microscopy image from the same spot.

When the hand-held resection probe is calibrated to the StealthStation, the coordinates at a specific measurement site are identified [27]. However, this is not enough to compensate for cerebral spinal fluid loss, and thus brain shift appears during the opening of the skull and removal of the dura. To minimize the impact of brain shift, an intraoperative or postoperative MRI is required. Following an intraoperative MR scan, the probe is recalibrated to the neuronavigation system and the surgery proceeds thereafter. By taking anatomical images into account, the fluorescence sites are better linked to the respective anatomical positions visible in the MRI. Furthermore, MRI protocols for diffusion and relaxometry can be added to the routinely captured T1- and T2-weighted anatomical images for a more detailed investigation of the relation between optical data, neuropathology, and MRI along a trajectory [31] or at the tumor marginal zone, which is a feature of specific interest for increasing the understanding of infiltrative brain tumors.

### 3.3. Needle Biopsy with Fluorescence Guidance

**Figure 6:**
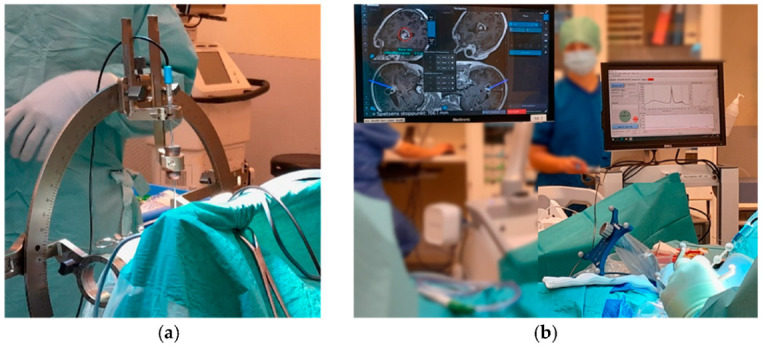
Brain tumor biopsy procedure using in situ optical measurement using the (**a**) Leksell Stereotactic System and the (**b**) StealthStation as a support system.

Figure 6 shows typical measurement situations in the neurosurgical OR during a needle biopsy when the LSS (Figure 6a) or the StealthStation (Figure 6b) is used as support system. As shown on the monitor (Figure 6b), real-time feedback is given for fluorescence, microcirculation, and tissue gray-whiteness as the biopsy kit with the probe inside progresses forward along the trajectory. This guides the surgeons to a position for tissue sampling. If several sites show high PpIX, the surgeons can use the Perfusion signal to avoid a site with higher blood flow to minimize bleeding when cutting the tissue at the sampling site.

Measurement through the biopsy needle during insertion resulted in fewer trajectories (28 in 27 patients) compared to the two-insertion procedure, where the optical probe was used to measure along the trajectory prior to insertion of the biopsy needle, which required 28 trajectories in 20 patients. The optical measurement time was 9 ± 6 min, with a range of 3–23 min. (n = 25), and the intraoperative pathology response time varied from 25 to 75 min, with an average of 44 ± 13 min. (n = 24). The optical measurement time was strongly dependent on the number of measurement sites and the time at each position, which was decided by the surgeon. In general, more sites and deeper positioned targets require longer measurement time. Fluorescence was seen in 43 out of the 47 patients. The final CNS WHO diagnosis was glioblastoma (n = 35), grade 2 glioma (n = 1), astrocytoma (n = 2), lymphoma (n = 7), and non-tumors (n = 2).

## 4. Discussion

Fluorescence spectroscopy using 5-ALA as a precursor has been proven to be beneficial for identifying high-grade tumor tissue like glioblastoma and lymphoma in situ during open surgery and burr hole needle biopsies. This paper summarizes studies with our optical systems and probes, presents our next generation of measurement system, and exemplifies practical use during surgery.

Needle biopsies are often afflicted with inconclusive results and complications such as hemorrhages [37,38,39]. Multiple trajectories may be required before tissue useful for pathology analysis is sampled. For each new insertion of the biopsy cannula along a trajectory, the risk for bleeding is increased and so is the total surgical time. With an optical probe inside the outer cannula modified with an opening at the tip, the light is transmitted to untouched tissue approximately 1 mm^3^ in front of the probe, where the optical information is collected. With real-time feedback, the optical signals support the surgeon in finding a suitable site for tissue sampling. In our study, only 1 of 27 patients required a second trajectory. Both trajectories showed PpIX peaks, but a second trajectory was required due to the inconclusive neuropathological answer from the first tissue sample. By implementing the intraoperative in situ measurement of PpIX fluorescence in combination with LDF, the diagnostic yield can increase, and the bleeding risks minimize. In this paper, we show that the total surgical time and number of trajectories are reduced. Further studies, including more clinics, would be warranted to verify the results on a larger scale. A comparative study of retrospective needle biopsies without optical measurements has been initiated and data will be compared with optically guided biopsies.

Other groups have also presented fluorescence spectroscopy systems but with the focus on resection [11]. Montcel et al. used tissue samples to evaluate their system and suggested a two-peak PpIX analysis method [40]. The same group evaluated their method in 10 patients in relation to resection [41]. Stummer and colleagues modified the FGR microscope and connected the backscattered light to optical fibers and a spectrometer during brain tumor resection [42]. It was used to evaluate the relation between visual inspection, PpIX spectra, and histopathology in 33 patients. Cornelius et al. introduced a mini-spectrometer [43] for clinical assessment in the surgeries of 29 metastases [17]. Recently, Lavrador et al., published a hand-held probe combining blue and white light spectroscopic measurements in relation to 35 resections [44]. Ndabakuranye et al. introduced a miniature system for PpIX fluorescence detection [45], which so far only has been evaluated in phantom and animal models, and not in a clinical situation that is more complex and demanding. In the OR aseptic conditions, electrical and laser safety needs to be under control and adapted to other equipment in the OR as well as to the clinical workflow.

The fluorescence system by Roberts and Valdes has been used in several resection studies enrolling at least 48 patients [46,47,48]. They also introduced an intraoperative probe for combined fluorescence and diffuse reflectance spectroscopy and used it in 10 patients during FGR [49]. With the goal to overcome the limitation of FGR in detecting tumor tissue at the marginal zone, a well-known weakness of FGR, they combined fluorescence with hyperspectral imaging on ex vivo biopsy samples [7]. In line with Stummer et al., [42] we have shown that a hand-held probe gives higher sensitivity along the marginal zone compared to FGR [29]. With the recent update of our system, FluoRa, the combination with microcirculation and gray-whiteness measurements will help to gain more insight into the brain tumor marginal zone. To our knowledge, no other group has combined LDF with fluorescence spectroscopy and fiber optical probe designs for resection and burr hole needle biopsies in clinical practice.

A well-known drawback with LDF is its sensitivity to movement artifacts. It is therefore essential to keep the probe still during the 5–10 s measurement. By using a navigation system or LSS, the biopsy needle with the probe inside is fixated while collecting data, and movement artifacts are thus reduced to a minimum. With the hand-held probe, movement artifacts can more easily interfere with the Perfusion signal. A general limitation with PpIX fluorescence guidance is the weak or lack of fluorescence in low grade tumors. In this paper, we have stressed the well-established, and FDA approved, use of microscopic fluorescence techniques as diagnostic markers of glioblastoma [2] and lymphoma [12,13]. PpIX fluorescence has also shown potential use in meningioma surgery [14,15] and for the identification of cerebral metastasis [16,17]. In our studies, no such cases are reported in adults. In our pediatric study, however, different tumor types were investigated, but only a few showed fluorescence [34], which also agrees with other studies [50,51,52]. Label-free methods such as Raman microscopy and spectroscopy are presently explored for identification of brain tumor tissue difficult to detect with PpIX fluorescence [8,9]. Another limitation is the necessity to administer 5-ALA orally prior to surgery, and the light sensitivity that can appear post-surgery. We have previously shown that the light sensitivity is reduced with a lower 5-ALA dose, without changing the diagnostic results [36]. Further studies will be required to confirm our findings before a decrease in dose can be recommended. Research is also ongoing to develop other precursors for fluorescence in detecting high-grade tumor tissue. One example is the synthetic 3-fluorinated 5-ALA analog [53].

Most of the suggested fluorescence spectrometer systems have been used in various experimental studies; only a limited number have reached the clinical setting. An excellent example of a successful translation for clinical use is the blue-light microscope. Many years of research were required before FGR was clinically accepted; the time to FDA approval was almost 20 years from the initial studies. Translating a system from an experimental setup to the investigational used in the clinic is a complex task that requires many careful steps. Electrical, mechanical, biological, and laser safety issues are some important aspects to consider. Others are the systems interaction with devices in the OR, and the training of the technical and clinical staff before data can be collected. It is not surprising that only a few groups have managed to take all steps towards clinical research use. Since the introduction of the Medical Device Directive in the EU, this process has become even more complex and demanding.

The Linköping systems have until now been evaluated in 75 tumor resections and 47 burr hole needle biopsies (Table 1). As a next step, we intend to introduce FluoRa 2.0 for investigational studies in both tumor resection and needle biopsies. Work is presently ongoing to obtain the system approved for clinical use. Furthermore, we intend to continue linking optical information and neuropathology to quantitative MRI methods such as relaxometry and diffusion imaging. The latter of which could increase knowledge on the microstructural level of the tumor marginal zone.

In conclusion, in more than 120 surgeries, we have shown that probe-based PpIX fluorescence spectroscopy is very useful for identification of glioblastoma and lymphoma in situ during brain tumor resection and burr hole needle biopsies. By integrating LDF with fluorescence spectroscopy in our FluoRa system, a new dimension for safer and more reliable tumor surgery is created. This is especially advantageous during needle biopsies, where in situ microcirculation and PpIX fluorescence measurements guide the surgeon to an optimal tissue sampling site.

## Figures and Tables

**Table 1:** Summary of studies at Linköping University Hospital using probe-based fluorescence spectroscopy measurements for detection of malignant brain tissue during resection and needle biopsies of brain tumors. 5-ALA = five aminolaevulinic acid; FGR = fluorescence-guided resection; FLS = fluorescence spectroscopy; LDF = laser Doppler flowmetry; LSS = Leksell Stereotactic System; N.A. = Not Applicable; Stealth = StealthStation; UL = Ultrasound Navigation.

Neurosurgical Procedure	No. Patients	Navigation	5-ALA [mg/kg]	Measurement Type	References
Tumor resection	1 3 ^1^	N.A. N.A.	5 5	mini-FLS FLS	[21] [22]
	9 ^1^ 30	UL N.A.	5 5/20	FLS FLS	[35] [36]
	16 14 ^2^ 5	N.A. N.A. Stealth	20 20 20	FLS/FGR FLS/FGR FluoRa 1.0/FGR	[29] [34] Not published
Tumor biopsy	20 3 20 4	LSS LSS Stealth Stealth	20 20 20 20	FLS/LDF FluoRa 1.0 ^3^ FluoRa 1.0 ^3^ FluoRa 1.0 ^3^	[25] [30] [31] Not published

^1^ same patients. ^2^ age 4–17. ^3^ probe inside modified biopsy needle.

## Data Availability

The data are not publicly available due to privacy regulations.
